# Cardiovascular ACE2 receptor expression in patients undergoing heart transplantation

**DOI:** 10.1002/ehf2.13528

**Published:** 2021-08-12

**Authors:** Johannes Bargehr, Patrick Rericha, Alex Petchey, Maria Colzani, Georgia Moule, Marie Chet Malgapo, Doris Rassl, Jason Tarkin, Greg Mellor, Fotis Sampaziotis, Teresa Brevini, Laure Gambardella, Martin R. Bennett, Sanjay Sinha

**Affiliations:** ^1^ Wellcome – MRC Cambridge Stem Cell Institute, Jeffrey Cheah Biomedical Centre, Cambridge Biomedical Campus University of Cambridge Cambridge UK; ^2^ Division of Cardiovascular Medicine University of Cambridge Cambridge UK; ^3^ Department of Histopathology Royal Papworth Hospital Cambridge UK; ^4^ Cardiology Department Royal Papworth Hospital Cambridge UK; ^5^ Department of Hepatology Cambridge University Hospitals NHS Foundation Trust Cambridge UK

**Keywords:** COVID‐19; SARS‐CoV‐2; Heart failure; Heart transplantation; ACE inhibitor

## Abstract

**Aims:**

Membrane‐bound angiotensin‐converting enzyme (ACE)2 is the main cellular access point for SARS‐CoV‐2, but its expression and the effect of ACE inhibition have not been assessed quantitatively in patients with heart failure. The aim of this study was to characterize membrane‐bound ACE2 expression in the myocardium and myocardial vasculature in patients undergoing heart transplantation and to assess the effect of pharmacological ACE inhibition.

**Methods and results:**

Left ventricular (LV) tissue was obtained from 36 explanted human hearts from patients undergoing heart transplantation. Immunohistochemical staining with antibodies directed against ACE2 co‐registered with cardiac troponin T (cTnT) and α‐smooth muscle cell actin (SMA) was performed across the entire cohort. ACE2 receptor expression was quantitatively assessed throughout the myocardium and vasculature. ACE2 was consistently expressed throughout the LV myocardium (28.3% ± 22.2% of cardiomyocytes). ACE2 expression was also detected in small calibre blood vessels (range, 2–9 μm), albeit at quantitatively much lower levels (5% ± 9% of blood vessels). There was no significant difference in ACE2 expression between patients receiving ACE inhibitors prior to transplantation and ACE inhibitor‐negative controls (*P* > 0.05). ACE2 expression did not differ significantly between the different diagnostic groups as the underlying reason for heart transplantation (ANOVA > 0.05). N‐terminal pro‐brain natriuretic peptide (NT‐proBNP) (*R* ^2^ = 0.37, *P* = 0.0006) and pulmonary capillary wedge pressure (PCWP) (*R* ^2^ = 0.13, *P* = 0.043) assessed by right heart catheterization were significantly correlated with greater ACE2 expression in cardiomyocytes.

**Conclusions:**

These data provide a comprehensive characterization of membrane‐bound cardiac ACE2 expression in patients with heart failure with no demonstrable effect exerted by ACE inhibitors.

## Introduction

Angiotensin‐converting enzyme 2 (ACE2) serves as a key counter‐regulator of the renin–angiotensin aldosterone system (RAAS). Decreased levels of ACE2 increase susceptibility to heart failure driven by the fibrotic, oxidative and pro‐inflammatory effects of the Ang II/AT_1_R axis, whereas increased ACE2 levels counterbalance these effects via the conversion of Ang II to Ang 1–7, imparting protection against heart failure.^1^ At the same time, membrane‐bound ACE2 and its accessory protease TMPRSS2 serve as the main cellular entry points for SARS‐CoV‐2, making them key determinants of disease susceptibility.^2^, ^3^

In the wake of the first global COVID‐19 wave, concerns were raised as to the safety of ACE inhibitors and angiotensin receptor blockers (ARBs), because preclinical data demonstrated that these drugs result in increased ACE2 expression.^4^, ^5^, ^6^ However, clinical studies have since shown that their use is safe in patients with COVID‐19 and does not result in increased disease susceptibility.^7^, ^8^, ^9^, ^10^ Indeed, ACE inhibitors and ARBs may exert protective effects as ACE2 is reduced in COVID‐19, resulting in unopposed detrimental action of AngII on the lungs.^11^

Cardiovascular complications occur in 20%–30% of patients critically ill with COVID‐19 and are harbingers of a complicated disease trajectory.^12^, ^13^ If the cardiovascular system is involved in the disease process, medical treatment can be complicated by cardiac arrhythmias, heart failure and myocardial injury.^12^, ^14^ One mechanism of myocardial injury is thought to be direct viral myocarditis, with ACE2 mediating viral entry. This is corroborated by in vitro studies and limited evidence from post‐mortem examinations as well as clinical studies investigating endomyocardial biopsies, demonstrating the presence of the SARS‐CoV‐2 genome in the myocardium.^15^, ^16^, ^17^, ^18^

Despite the safety of ACE inhibitors and ARB use in large cohorts of patients with COVID‐19, specific subgroups of patients with particularly high cardiac ACE2 expression may be at increased risk of myocarditis if pharmacological ACE inhibition use further increases cardiac membrane‐bound ACE2 levels. In heart failure, it has been demonstrated that soluble ACE2 levels are increased, but there is a paucity of data on the effects of ACE inhibitors on the expression of membrane‐bound cardiac ACE2 in heart failure.^19^ Indeed, most data on cardiac ACE2 protein expression come from rodents, demonstrating expression in cardiomyocytes, smooth muscle cells, endothelial cells and cardiac fibroblasts.^20^, ^21^, ^22^

In non‐diseased human hearts, single‐cell sequencing has demonstrated that 6.6% of cardiomyocytes express ACE2.^23^ Single‐nucleus RNA sequencing of human hearts has shown increased ACE2 expression in cardiomyocytes and decreased expression in pericytes, smooth muscle cells and fibroblasts of patients with dilated cardiomyopathy and hypertrophic cardiomyopathy compared with normal hearts.^24^ In line with these findings, bulk sequencing of failing hearts has demonstrated increased ACE2 mRNA expression and increased protein expression compared with non‐diseased hearts.^25^ However, major drawbacks of such transcriptional measurements include that they do not take into account RNA half‐life, translation efficacy or protein turnover. This highlights that studies investigating membrane‐bound ACE2 protein are essential, particularly in vulnerable groups.

Here, we demonstrate the quantitative assessment of ACE2 expression across the explanted left ventricular (LV) myocardial tissues of 36 heart transplant recipients. We elucidate expression in cardiomyocytes and the blood vasculature and assess the effect of ACE inhibitor administration prior to transplantation. Furthermore, we provide corollary data on clinical characteristics and myocardial ACE2 receptor expression. These data serve as an important source of validation for sequencing studies and broaden the current insight into ACE2 expression in human hearts.

## Methods

### Study design and population

**Figure 1:**
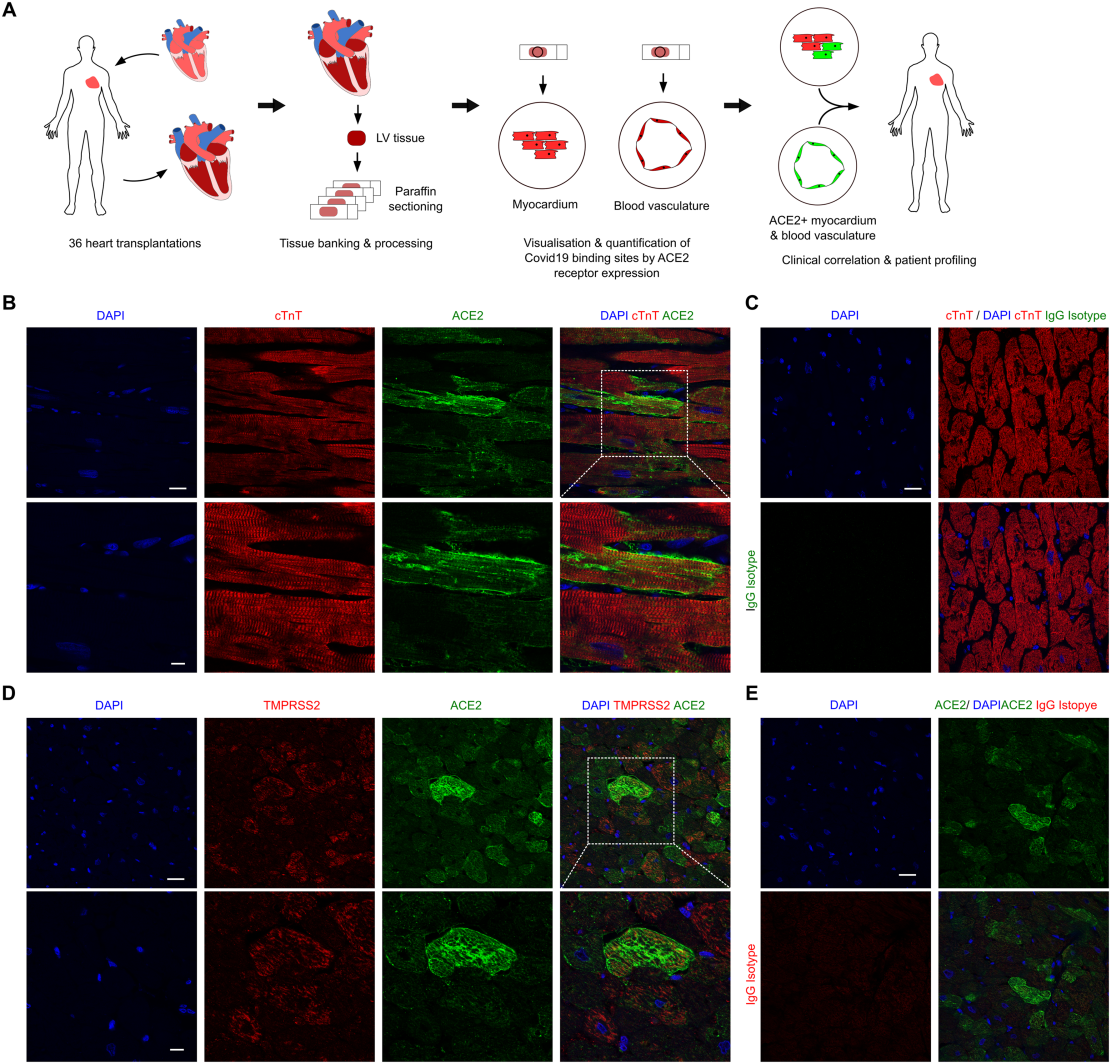
Visualization of Covid19 binding sites by ACE2 receptor expression. (A) Schematic of study design. Heart explant sections were obtained from 36 patients undergoing heart transplantation. Paraffin‐embedded sections were stained for cTnT/ACE2 and SMA/ACE2 across the entire cohort. The results were subsequently correlated with pretransplant characteristics. (B) Validation of ACE2 antibodies. Expression of ACE2 in human left ventricular myocardium. Scale bars: 25 and 10 μm, respectively. (C) IgG Isotype control for ACE2, demonstrating specificity of staining. Scale bar, 25 μm. (D) Expression of the accessory protease TMPRSS2 and ACE2, demonstrating co‐expression in a subset of cardiomyocytes. Scale bars: 25 and 10 μm, respectively. (E) IgG Isotype control for TMPRSS2, demonstrating specificity of staining. Scale bar: 25 μm.Abbreviations: LV, left ventricular; ACE2, angiotensin‐converting enzyme 2; cTnT, cardiac muscle troponin T; IgG, immunoglobulin G.

This study was designed to investigate the expression of ACE2 receptor expression across explanted human hearts and determine the effect of pharmacological ACE inhibition. The study design is depicted in *Figure* *1* *A*. Tissues from explanted hearts of 36 patients who underwent heart transplantation at Royal Papworth Hospital for end‐stage heart failure between 5 August 2013 and 28 December 2018 were sourced from the Royal Papworth Hospital tissue bank. Cases were selected if 4% formalin‐fixed and paraffin‐embedded LV tissue blocks were available. Cases were classified as dilated cardiomyopathy (DCM), hypertrophic cardiomyopathy (HCM), ischaemic cardiomyopathy (ICM) or arrhythmogenic cardiomyopathy (ACM) and were categorized into ACE inhibitor administration present or absent based on the history. Cases with a more recent inclusion date were selected first. We attempted to select an equal number of cases established on ACE inhibitors and ACE inhibitor‐negative controls. Equally we included the same number of cases per underlying cardiac aetiology in the ACE inhibitor groups and the ACE inhibitor‐negative control group.

The clinical baseline characteristics of the cohort are shown in *Table* *1*. Tissue blocks were sectioned, and immunohistochemistry, imaging, quantification and statistical analysis were performed. The only exclusion criterion was administration of ARBs as this is a potential confounder of ACE2 receptor expression. Ethical approval to provide human tissue for research was granted to the Royal Papworth Hospital (RPH) Research Tissue Bank by the East of England—Cambridge East Research Ethics Committee (REC reference: 18/EE/0269). Participants whose explanted hearts were donated to the RPH tissue bank had provided written, informed consent.

**Table 1:** Baseline characteristics of 36 patients undergoing heart transplantation

Variable	All Patients (*n* = 36)
Age, years, mean ± SD	51 ± 13 (54; 18–82)
Male, sex, *n* (%), male	26 (72%)
Race, *n* (%)	
White—British	32 (89%)
Black or black British	2 (6%)
Any other ethnic background	2 (6%)
Underlying diagnosis for HTx, *n* (%)	
Hypertrophic cardiomyopathy	12 (33%)
Dilated cardiomyopathy	8 (22%)
Ischaemic heart disease	8 (22%)
Arrhythmogenic cardiomyopathy	8 (22%)
Listing state, *n* (%)	
Non‐urgent	17 (49%)
Urgent	18 (51%)
NYHA functional class, *n* (%)	
NYHA 1	1 (4%)
NYHA 2	1 (4%)
NYHA 3	21 (75%)
NYHA 4	5 (18%)
6MWT distance (m), mean ± SD	314.1 ± 96.9 (336; 140–452)
CPEX − VO2max (mL/kg/min), mean ± SD	14.3 ± 7.01 (12.2; 5.8–40)
ECG, *n* (%)	
QRS > 120 ms	19 (53%)
LBBB	4 (11%)
RBBB	6 (17%)
Inotropic support, *n* (%)	11 (31%)
Device therapy, *n* (%)	27 (75%)
ICD	17 (47%)
BiV pacemaker	7 (19%)
BiV ICD	10 (28%)
Impella	0 (0%)
IABP	3 (8%)
LVAD	5 (14%)
ECMO	1 (3%)
Coexisting disorders and interventions, *n* (%)	
Hypertension	6 (17%)
Diabetes	7 (19%)
Atrial fibrillation	18 (50%)
Atrial flutter	7 (19%)
Stroke	6 (17%)
Coronary artery disease	3 (8.3%)
Myocardial infarction	8 (23%)
Previous PCI	6 (17%)
Previous CABG	2 (6%)
COPD	2 (6%)
Chronic lung disease	4 (11%)
Active smoker	4 (11%)
Chronic renal disease	8 (22%)
Pretransplant cardiovascular drug therapy, *n* (%)	
ACE inhibitor	19 (53%)
Time on ACE inhibitor (days)	570 ± 768 (349; 47 to 3,073)
Beta‐blockade	25 (70%)
Aldosterone blocker	25 (70%)
Furosemide	16 (44%)
Bumetanide	11 (31%)
Metolazone	1 (3%)
Physiologic parameters prior to HTx, mean ± SD	
Right heart catheter	
PCWP (mmHg)	17 ± 6.3 (17.5; 1–28)
RA pressure (mmHg)	11.2 ± 6.5 (10; 1–26)
CI (l/min/m^2^)	1.69 ± 0.42 (1.6; 1.1–2.8)
Echo	
LVEF (%)	27.5 ± 15.2 (20; 6–60)
TAPSE (mm)	14.3 ± 4.3 (14; 6.1–25)
sPAP (mmHg)	39 ± 17.9 (40; 1–77)
LVESD (mm)	40.4 ± 16 (38; 4.7–71)
LVEDD (mm)	54.3 ± 14.9 (56; 5.6–79)
Laboratory data, mean ± SD	
NT‐proBNP (pg/mL)	4,453 ± 3,155 (3,355; 157–12,444)
Serum creatinine (μmol/L)	119 ± 35 (111.5; 58–189)
Na (mmol/L)	137.4 ± 4.2 (138; 125–143)
Hb (g/L)	120 ± 43 (130; 12.7–159)
Bilirubin (μmol/L)	23.69 ± 12.8 (20; 7–62)
ALT (U/L)	50.2 ± 61.5 (37; 18–345)
Albumin (g/L)	61.58 ± 79.2 (42.5; 35–436)

6MWT, 6‐min walk test; ALT, alanine aminotransferase. BiV, biventricular; CABG, coronary artery bypass grafting; CI, cardiac index; COPD, chronic obstructive lung disease; CPEX, cardiopulmonary exercise testing; ECMO, extracorporeal membrane oxygenation; Hb, haemoglobin; ICD, implantable cardioverter defibrillator; LBBB, left bundle branch block; LVAD, left ventricular assist device; LVEDD, left ventricular end‐diastolic dimension; LVEF, left ventricular ejection fraction; LVESD, left ventricular end‐systolic dimension; NT‐pro BNP, N‐terminal prohormone of brain natriuretic peptide; NYHA, New York Heart Association; PCI, percutaneous coronary intervention; PCWP, pulmonary capillary wedge pressure; RA, right atrial; RBBB, right bundle branch block; SPAP, systolic pulmonary artery pressure; TAPSE, tricuspid annular plane systolic excursion.

### Tissue processing and immunohistochemistry

LV tissues were consistently collected from the lateral free wall, fixed in 4% paraformaldehyde, paraffin‐embedded, sectioned and additionally wax‐dipped to optimize preservation. For immunohistochemistry, slides were dewaxed for 20 min at 60°C and subsequently deparaffinized, before being subject to heat‐mediated antigen retrieval with citric acid buffer for 10 min with pH optimized at 6 as previously described.^26^ Slides were subsequently blocked with 5% BSA/PBS containing 0.3% Triton X‐100 for 1 h at room temperature. This was followed by incubation with primary antibodies at 4°C overnight and application of fluorescent secondary antibodies at room temperature for 60 min on the consecutive day. A detailed description of the antibodies and dilutions used is provided in *Table* S1.

### Imaging

All qualitative images shown were acquired on a Leica SP5 confocal microscope using Leica imaging software. For quantitative image analysis, three representative images were acquired across all 36 patients on an EVOS FL digital inverted microscope (Life Technologies) for both ACE2/cTnT and ACE2/α‐smooth muscle cell actin (SMA) co‐staining.

### Quantification and validation

**Figure 2:**
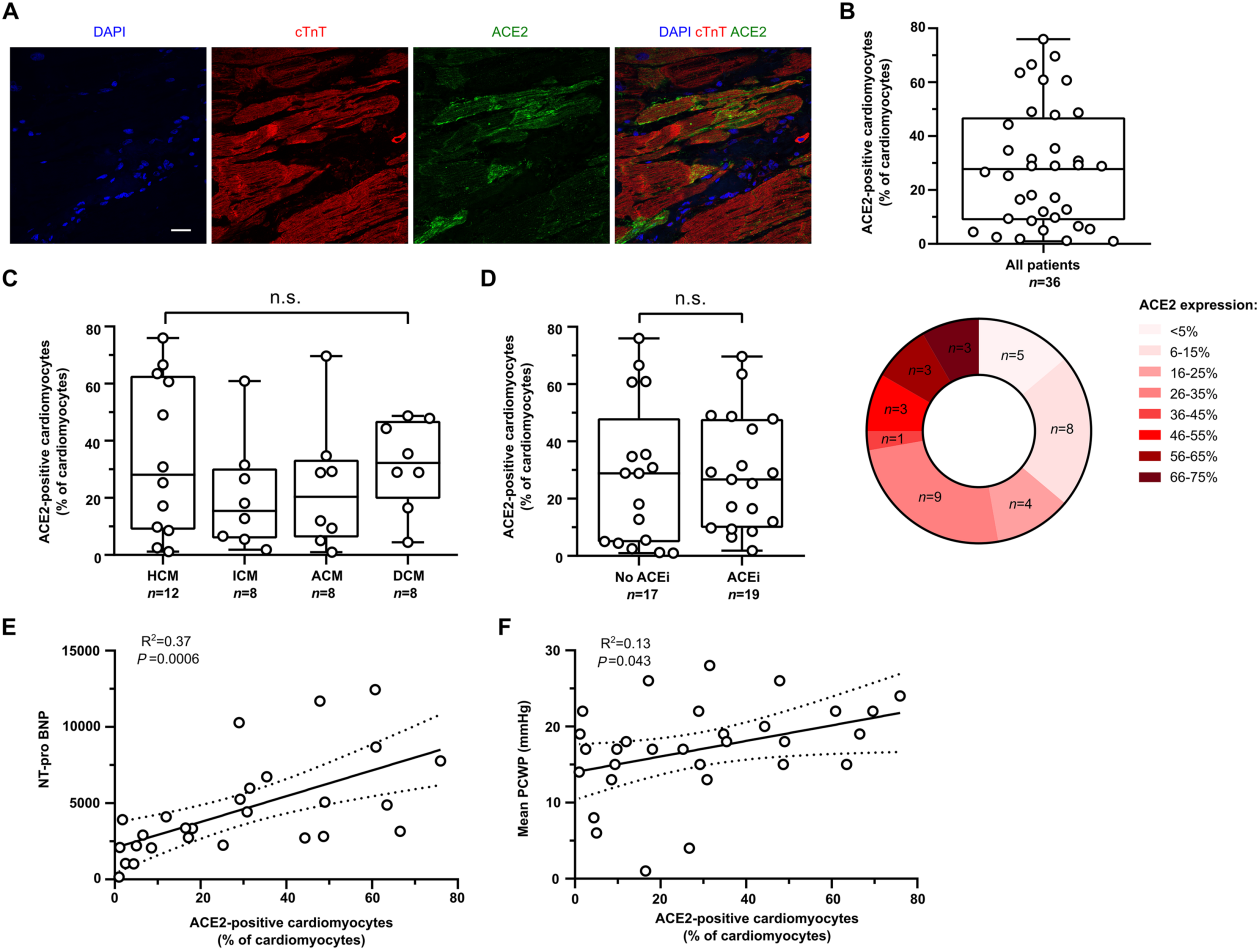
Quantification of myocardial ACE2 expression in explanted human hearts.(A) ACE2 expression in human cardiomyocytes in explanted hearts. Scale bar: 25 μm. (B) Quantification of ACE2 expression expressed as % of ACE2‐positive cardiomyocytes of all cardiomyocytes. Bottom pie chart shows group stratification. (C) ACE2 expression in patients according to underlying reason for heart transplantation. HCM, hypertrophic cardiomyopathy; ICM, ischaemic cardiomyopathy; ACM, arrhythmogenic cardiomyopathy; DCM, dilated cardiomyopathy. (D) Effect of ACE inhibitor administration on ACE2 expression. (E) Baseline clinical variables significantly correlated with myocardial ACE2 expression. Shown is NT‐proBNP. (F) Correlation of PCWP (mmHg) with myocardial ACE2 expression.Abbreviations: ACE2, angiotensin‐converting enzyme 2; cTnT, cardiac muscle troponin T; IgG, immunoglobulin G.

Image quantification was performed with ImageJ. For quantification of ACE receptor expression across the myocardium, sections stained with antibodies directed against ACE2 and cTnT were imported, and the number of cardiomyocytes was manually counted, followed by counting of ACE2‐positive cardiomyocytes for which preset rules were applied. Cells were identified as cardiomyocytes if a nucleus could be clearly attributed to the surrounding cTnT signal. Binucleated cells were counted as one cardiomyocyte. All cardiomyocytes were counted on all 108 images (three technical replicates per patient) amounting to 10 707 cardiomyocytes identified in total. If there was expression of ACE2 in previously identified cardiomyocytes, this was expressed as ACE2 + cardiomyocytes (% of all cardiomyocytes identified). For quantification of ACE2 expression across the vasculature, sections stained with antibodies directed against ACE2 and SMA were analysed. Structures with a clearly identifiable lumen and SMA positivity were identified as blood vessels and numerically assessed. The number of ACE2 expressing blood vessels was counted, and the diameter of ACE2 positive blood vessels was measured. *Figures* *2* *B* *and* *3* *B* show a circular heatmap displaying a group stratification based on the % of cardiomyocytes and % of blood vessels, respectively, accounting for ACE2 expression, and show the number of patients falling into each group. *Table* S2 shows all clinical characteristics correlated with ACE2 expression in cardiomyocytes and blood vessels, respectively.

All images were anonymized, and a primary reader made measurements in a blinded manner. For validation purposes, a sample set of images was analysed by an independent investigator in a blinded fashion to ensure reproducibility in measurements. The respective Bland–Altman plots and intraclass correlation coefficients of these two tests are presented in *Figure* S1.

### Statistical analysis

All analysis of quantitatively acquired images was performed in three technical replicates per patient (i.e. three images per patient). All experimental data specifically state the number of patients assessed for quantitative endpoint analysis. The normal distribution of our values was confirmed using the D'Agostino and Pearson omnibus normality test where appropriate. Statistical testing was performed using an unpaired *t*‐test for two‐group comparisons, and a one‐way ANOVA with a post hoc Tukey test was used for multiple‐group comparison. Measuring two‐sided significance, a *P‐*value of 0.05 was considered statistically significant. All analyses were performed using GraphPad Prism software in a blinded fashion. All results are expressed as mean ± SD (median; min to max), unless otherwise stated.

## Results

### Study population

A total of 36 patients who underwent heart transplantation between August 2013 and December 2018 for end‐stage heart failure were included in the study. The clinical baseline characteristics are depicted in *Table* *1*. Mean patient age was 51 years ± 13 (54; 18–82) with 26 patients (72%) being male. Pharmacological ACE inhibition was present in 19 patients (53%) with the remainder serving as ACE inhibitor‐negative controls. ARB administration was an exclusion criterion to avoid confounding on ACE2 expression. Underlying reason for transplantation included HCM (*n* = 12), DCM (*n* = 8), ICM (*n* = 8) and ACM (*n* = 8). Listing state was urgent in 18 patients (51%), and NYHA functional Class 3 was the most common class, recorded in 21 patients (75%). The mean 6MWTD and cardiopulmonary exercise testing accounted for 314 ± 96 m and for a VO2max of 14.3 ± 7, respectively. Pretransplant inotropic support was required in 11 patients (31%), and 27 patients (75%) required some sort of device therapy. Right heart catheterization demonstrated mean PCWP, RAP and CI values of 17 ± 6.3 mmHg, 11.2 ± 6.5 mmHg, and 1.69 ± 0.42 l/min/m^2^, respectively. Echo accounted for mean LVEF and TAPSE of 27.5 ± 15.2% and 14.3 ± 4.3 mm, respectively. Mean NT‐proBNP was 4592 ± 3231 pg/mL.

### Expression and validation of ACE2 in explanted human hearts

*Figure* *1* *A* depicts the workflow and design of the study. The LV tissue blocks of 36 patients having undergone heart transplantation were retrieved, sectioned and subjected to immunohistochemical staining. LV tissue sections were stained with antibodies directed against ACE2 and cTnT as well as ACE2 and SMA and were subsequently quantified and correlated with clinical baseline characteristics.

*Figure* *1* *B* visualizes ACE2 expression in explanted human LV myocardium. Specificity of the staining is confirmed by neighbouring cardiomyocytes being ACE2 negative compared with others that account for a strongly positive signal. Specific ACE2 staining is further corroborated by a lack of signal upon IgG isotype staining (*Figure* *1* *C*). Because COVID‐19 uses both ACE2 and the accessory protease TMPRSS2 to gain access to human cells, we confirmed co‐expression of ACE2 and TMPRSS2 in human cardiomyocytes (*Figure* *1* *D*). Specificity of TMPRSS2 staining is demonstrated by positivity in some cardiomyocytes and lack of expression in others. This is furthermore readily shown by a lack of signal upon IgG isotype staining.

In summary, heterogeneous ACE2 expression can be robustly visualised by specific immunohistochemical staining, and a proportion of cardiomyocytes co‐expresses ACE2 and the accessory protease TMPRSS2.

### ACE2 expression in human LV myocardium

To demonstrate expression of ACE2 across the LV myocardium, all ACE2‐expressing cardiomyocytes were quantitatively assessed by manual counting in a blinded fashion across three images per patient. For validation purposes and to ensure reproducibility, this was confirmed by a second independent investigator. The respective Bland–Altman plots and intraclass correlation coefficients are shown in *Figure* S1, confirming sound interobserver variability within a preset range of 4%.

Quantitative assessment of ACE2 expression in cardiomyocytes demonstrated that 28.3% ± 22.2% of LV cardiomyocytes expressed ACE2. Indeed, robust ACE2 expression in LV myocardium was seen in the majority of patient samples examined. Group stratification demonstrated that ACE2 cardiomyocyte positivity was >5% in 31 patients (86%), >25% in 19 patients (52%) and >45% in 9 patients (25%) (*F* *igure* *2* *A* *and* *2* *B*).

There were no significant differences between ACE2 expression in cardiomyocytes (%) across the four different diagnostic groups, including HCM, ICM, ACM or DCM (ANOVA *P* > 0.05) (*Figure* *2* *C*). Importantly, we did not detect a significant difference in the % of ACE2‐expressing cardiomyocytes in ACE inhibitor‐treated patients compared with ACE inhibitor‐negative patients (*Figure* *2* *C* *and* *2* *D*).

We next sought to identify clinical characteristics present prior to transplantation that could predict ACE2 expression in cardiomyocytes. Correlation analysis demonstrated that NT‐proBNP is significantly positively correlated with % of ACE2‐expressing cardiomyocytes (*R* ^2^ = 0.37, *P* = 0.0006). PCWP obtained from right heart catheterization also had a significant positive correlation with ACE2 expression in cardiomyocytes (*R* ^2^ = 0.13, *P* = 0.043).

Taken collectively, quantitative analysis demonstrated robust expression of ACE2 in the myocardium of the majority of patients. Administration of ACE inhibitors had no effect on ACE2 expression, but greater levels of NT‐proBNP and PCWP were correlated with greater ACE2 expression in cardiomyocytes.

### ACE2 expression in the blood vasculature

**Figure 3:**
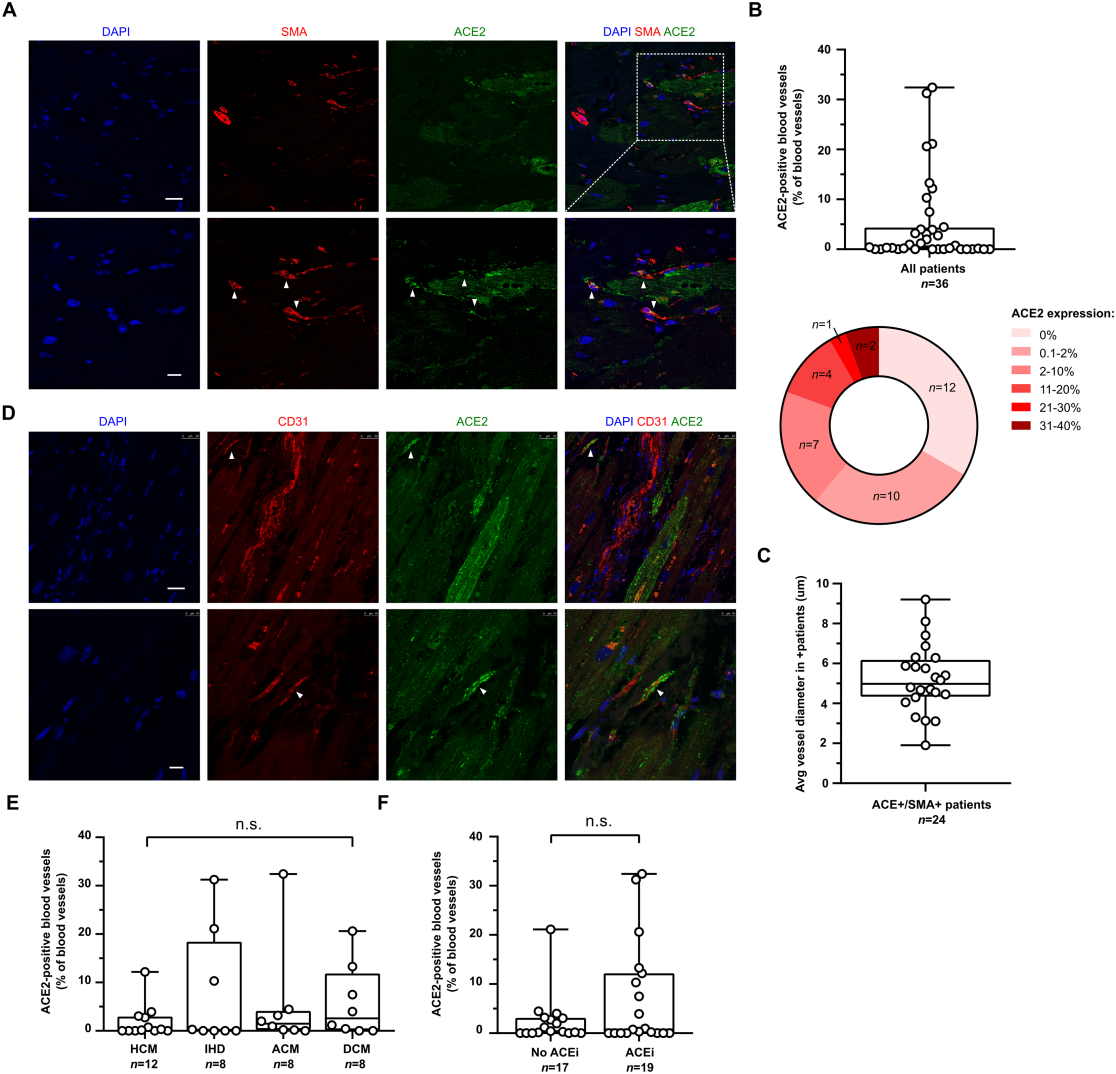
Quantification of vascular ACE2 expression in explanted human hearts.(A) ACE2 and SMA co‐expression in blood vasculature of explanted human hearts. White arrows indicate co‐expression. Scale bars: 25 and 10 μm, respectively. (B) Quantification of ACE2 expression expressed as % of ACE2‐positive blood vessels of all blood vessels. Bottom pie chart shows group stratification. (C) Average vessel diameter in μm in ACE2+/SMA+ patients. (D) ACE2 and CD31 co‐expression in blood vasculature of explanted human hearts. White arrow indicates co‐expression. (E) Vascular ACE2 expression in patients according to underlying reason for heart transplantation. HCM, hypertrophic cardiomyopathy; ICM, ischaemic cardiomyopathy; ACM, arrhythmogenic cardiomyopathy; DCM, dilated cardiomyopathy. (F) Effect of ACE inhibitor administration on ACE2 expression in blood vasculature.Abbreviations: SMA, alpha smooth muscle actin; ACE2, angiotensin‐converting enzyme 2; CD31, cluster of differentiation 31; ACEi, angiotensin‐converting enzyme inhibitor.

To demonstrate ACE2 expression across the vasculature, slides were stained with antibodies directed against ACE2 and SMA. SMA is expressed by smooth muscle cells in the arteriolar wall as well as by pericytes residing in the wall of the microcirculation. Qualitative assessment of ACE2‐expressing SMA‐positive blood vessels demonstrated a mean value of 5% ± 9% (1%; 0%–32%). In contrast to expression seen in cardiomyocytes, ACE2 was not detected at all in the blood vessels of 12 patients (33%) as demonstrated by the corresponding heatmap (*Figure* *3* *A* *and* *3* *B*). Another 10 patients (28%) showed ACE2 expression in only 0.1%–2% of all SMA + blood vessels. The remainder (*n* = 14) demonstrated expression above 2% of all SMA‐expressing vessels, with seven patients (19%) demonstrating ACE2 expression in 2%–10% and another seven patients (19%) showing expression in 11%–40% of all SMA + blood vessels detected.

To further characterize the size of ACE2 positive blood vessels, the diameter of positive vessels was measured. This demonstrated a range of vessel diameters from 2 to 9 μm, demonstrating that in this analysis, ACE2 was not expressed by large calibre blood vessels (*Figure* *3* *C*). We have additionally performed immunohistochemical staining with antibodies directed against ACE2 and CD31 and confirmed co‐expression in a small subset of blood vessels (*Figure* *3* *D*).

In line with our analysis of ACE2 expression in cardiomyocytes, we also did not detect a significant difference in ACE2 expression in blood vessels between the four different diagnostic groups (ANOVA *P* > 0.05). Furthermore, there was no significant difference in ACE2 expression in SMA + blood vessels between patients on pharmacological ACE inhibition and ACE inhibitor‐negative patients (*Figure* *3* *E* *and* *3* *F*).

In conclusion, ACE2 expression can be found in blood vessels with a diameter ranging from 2 to 9 μm but remains largely below the % expression levels seen in cardiomyocytes.

## Discussion

This study was designed to investigate ACE2 receptor protein distribution in a quantitative fashion throughout human LV myocardium and the vasculature in to date the largest series of its kind. Here, we demonstrated that ACE2 co‐localizes with the accessory protease TMPRSS2 in LV myocardium and provide a detailed characterization of its distribution throughout cardiomyocytes, demonstrating marked expression in this severely diseased cohort. The percentage of expression in cardiomyocytes was correlated with NT‐proBNP and PCWP, two markers of increased intraventricular pressures. In contrast, the extent of expression in the blood vessels was markedly lower and confined to small calibre vessels. Importantly, the administration of pharmacological ACE inhibition did not affect membrane‐bound ACE2 receptor expression at the tissue level.

Although heart failure results in an increase in plasma ACE2, which predicts disease severity and poor outcome, few data are available on membrane‐bound ACE2.^19^ Similarly, in the general population, ACE2 levels predict all‐cause and cardiac mortality with no demonstrable effect exerted by ACE inhibitors and ARBs on soluble ACE2 expression.^27^ In view of the risk of myocarditis in the ongoing COVID‐19 pandemic, it is essential to understand expression of membrane‐bound ACE2 in vulnerable subgroups such as heart failure patients and to assess the effects of pharmacological ACE inhibition.

A recent study by Donoghue and colleagues demonstrated ACE2 protein expression by immunohistochemistry in endothelial cells and smooth muscle cells only with no difference seen in failing and non‐diseased hearts.^28^ However, the number of samples was not stated in this study, which is of particular importance as our data demonstrate that vascular expression of membrane‐bound ACE2 is heterogeneous in this cohort, with few demonstrating high expression and a third with no detectable signal. Furthermore, it is unclear why ACE2 was not detected in cardiomyocytes by Donoghue and colleagues, although in our study we used a different primary antibody and a highly sensitive immunofluorescence protocol.

Single‐cell sequencing data are poorly suited for a comparison with protein data but suggest that only a subset of cardiomyocytes express ACE2. In non‐diseased human hearts, 6.6% of cardiomyocytes expressed ACE2 mRNA.^23^ In contrast, in a study by Tucker and colleagues, single‐cell nucleus RNA sequencing demonstrated increased ACE2 expression in patients with DCM and HCM compared with patients with non‐diseased hearts.^24^ There was a non‐significant trend for increased ACE2 expression with pharmacological ACE inhibition. Nicin and colleagues demonstrated by single‐cell sequencing that cardiomyocyte ACE2 mRNA expression is increased in aortic stenosis and heart failure.^29^ This study furthermore found increased ACE2 expression with administration of ACE inhibitors but not with ARBs. Bulk sequencing data demonstrated increased ACE2 mRNA expression in failing hearts compared with non‐diseased hearts with the drawback being that it remains unclear which cell types account for this effect.^25^ Taken collectively, these sequencing studies highlight the need to clarify protein responses to validate how these findings translate from the mRNA level to the protein level and to elucidate protein expression in the respective cell types.

Of note, transcriptomic studies have demonstrated high levels of ACE2 in pericytes and vascular smooth muscle cells. Our data demonstrate detectable protein expression of membrane‐bound ACE2 in small calibre, pericyte‐containing blood vessels in two‐thirds of the patients investigated (*n* = 24). However, ACE2 expression was present to a much lesser degree in the vasculature than seen throughout the LV in cardiomyocytes, highlighting the need for confirmatory testing in larger series of patient tissues to put these sequencing findings into perspective. The co‐expression of ACE2 and TMPRSS2 in cardiomyocytes seen in this study supports the notion that COVID‐19 can gain access and cause direct cytotoxic damage to cardiomyocytes, which is supported by post‐mortem and clinical studies as well experimental data.^15^, ^16^, ^18^

The clinical sequelae of SARS‐CoV‐2 are well known to also include vascular complications that are thought to occur at least in part due to direct viral cytotoxic damage exerted on the endothelium.^30^, ^31^, ^32^, ^33^ In line with this, post‐mortem studies have identified microthrombi as a key pathologic contributor to myocardial necrosis.^34^ Our data demonstrate ACE2 expression in the LV vasculature, quantitatively in smooth muscle cells and qualitatively in endothelial cells. In this context, we have previously demonstrated that hESC‐derived smooth muscle cells can be accessed by SARS‐CoV‐2 but do not act as a reservoir for replication.^17^ The combination of direct endothelial damage and cytotoxicity is likely to result in the clinical complications seen, including thromboembolic disease, AKI and neurological disorders.^31^, ^35^

Finally, our data demonstrate that serum NT‐proBNP and PCWP assessed by right heart catheterization, both markers of increased intramyocardial pressures, predict greater ACE2 expression in cardiomyocytes. In this context, DCM and HCM have previously been demonstrated to be associated with increased ACE2 expression in LV cardiomyocytes.^24^, ^36^ This is in line with recent data demonstrating that an increase in remodelling in patients with DCM results in increased ACE2 expression with reverse remodelling reversing this process.^37^ It is conceivable that the beneficial effects of ACE2 via Ang 1–7 reach a ceiling while ACE2 expression continues to increase. Although this warrants investigation in a prospective setting, as certain patients with end‐stage heart failure exhibited particularly high levels of ACE2 expression, this could suggest a greater susceptibility to COVID‐19.

COVID‐19 results in grave cardiovascular morbidity and mortality in patients with heart failure. Our study suggests that some of this increased risk may be due to direct interaction between SARS‐CoV‐2 and cardiomyocytes given the high expression of membrane‐bound ACE2 in cardiomyocytes and the observed co‐expression with TMPRSS2. ESC/AHA/ACC guidelines for this group of patients suggest to continue treatment with ACE inhibitors and ARBs and our data are in support of this because no upregulation of membrane‐bound ACE2 with pharmacological ACE inhibition was demonstrated. Whether greater levels of membrane‐bound ACE2 translate to greater COVID‐19 disease susceptibility warrants investigation in a prospective setting. Finally, this dataset serves as an important source of validation for ongoing sequencing studies.

## Conflict of interest

The authors have nothing to disclose.

## Funding

This work was supported by a British Heart Foundation Centre for Cardiovascular Research Excellence Covid‐19 pump‐priming award RE/18/1/34212 and a BHF Senior Fellowship FS/18/46/33663 (L.G., S.S.). S.S. was also supported by the British Heart Foundation Centre for Cardiovascular Research Excellence. M.C. is supported by CRM (RM/17/2/33380) and also has support from BHF grant SP/15/7/31561. We also acknowledge core support from the Wellcome Trust and MRC to the Wellcome Trust – Medical Research Council Cambridge Stem Cell Institute. This research was funded in whole, or in part, by the Wellcome Trust [203151/Z/16/Z] and the UKRI Medical Research Council [MC_PC_17230]. We also acknowledge support from the NIHR Cambridge Biomedical Research Centre (BRC‐1215‐20014). For the purpose of Open Access, the author has applied a CC BY public copyright licence to any Author Accepted Manuscript version arising from this submission.

## Supporting information


**Figure S1.** Validation of quantitative immunohistochemical analysis. A) Inter‐observer agreement of quantitative assessment of ACE2 + cardiomyocytes. B) Inter‐observer agreement of quantitative assessment of ACE2 + blood vessels. For both A) and B) Bland–Altman plots exhibits the mean difference as well as the difference in % of ACE2 + CMs/blood vessels between the independent measurements of two blinded investigators. The interobserver agreement was compared for 8 randomly selected images for each of % of ACE2 + cardiomyocytes (n = 8) and ACE2 + blood vessels (n = 8). The dotted line shows the mean difference of all readouts and the continuous lines exhibit the a priori agreed limits of allowed deviation in measurements (i.e. 5%). The Intraclass correlation coefficient (ICC) is shown for all measurements and the corresponding 95%CI in parenthesis.

**Table S1.** Primary and secondary antibodies used for IHC.

**Table S2.** Clinical variables significantly correlated with cardiac ACE2 expression or showing a non‐significant trend.
Abbreviations: NT‐pro BNP, N‐terminal prohormone of brain natriuretic peptide; PCWP, pulmonary capillary wedge pressure; LVESD, left ventricular end‐systolic dimension; BSA, body surface area; CPEX, cardiopulmonary exercise testing; ECMO, extracorporeal membrane oxygenation; CABG, coronary artery bypass grafting; AF, atrial fibrillation.
