# Supplementary figures and images for: Cardiovascular ACE2 receptor expression in patients undergoing heart transplantation
Source: ESC Heart Fail. 2021 Aug 12;8(5):4119–29. doi: 10.1002/ehf2.13528 (PMC8497226; doi:10.1002/ehf2.13528)

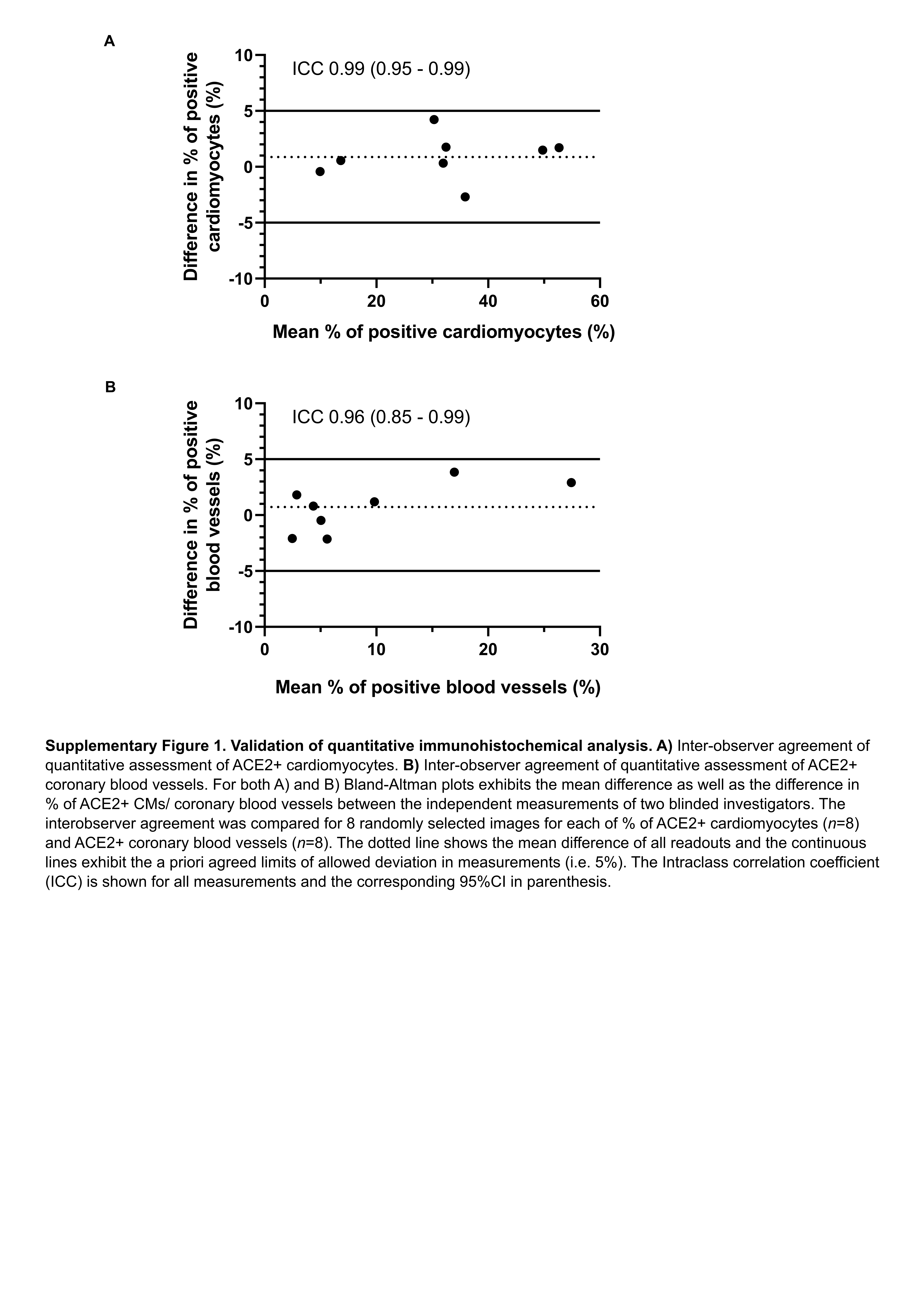

Supplement: Supplementary file 1 — Figure S1. Validation of quantitative immunohistochemical analysis. A) Inter‐observer agreement of quantitative assessment of ACE2 + cardiomyocytes. B) Inter‐observer agreement of quantitative assessment of ACE2 + blood vessels. For both A) and B) Bland–Altman plots exhibits the mean difference as well as the difference in % of ACE2 + CMs/blood vessels between the independent measurements of two blinded investigators. The interobserver agreement was compared for 8 randomly selected images for each of % of ACE2 + cardiomyocytes (n = 8) and ACE2 + blood vessels (n = 8). The dotted line shows the mean difference of all readouts and the continuous lines exhibit the a priori agreed limits of allowed deviation in measurements (i.e. 5%). The Intraclass correlation coefficient (ICC) is shown for all measurements and the corresponding 95%CI in parenthesis. [file EHF2-8-4119-s003.png]
