# Supplementary material for: Cardiovascular ACE2 receptor expression in patients undergoing heart transplantation
Source: ESC Heart Fail. 2021 Aug 12;8(5):4119–29. doi: 10.1002/ehf2.13528 (PMC8497226; doi:10.1002/ehf2.13528)
Supplement: Supplementary file 2 — Table S1. Primary and secondary antibodies used for IHC. [file EHF2-8-4119-s002.docx]

| **Antibody** | **Application** | **Species** | **Dilution** | **Manufacturer (Cat#)** |
| --- | --- | --- | --- | --- |
| ACE2 | IHC | Rabbit | 1:200 | Abcam (ab15348) |
| ACE2 | IHC | Goat | 1:200 | R&D (AF933) |
| TMPRSS2 | IHC | Rabbit | 1:200 | Abcam (ab109131) |
| Cardiac Troponin T | IHC | Goat | 1:200 | Abcam (ab64623) |
| Cardiac Troponin T | IHC | Mouse | 1:200 | Abcam (ab8295) |
| Smooth Muscle Alpha Actin | IHC | Mouse | 1:200 | Dako (M0851) |
| CD31 | IHC | Rabbit | 1:200 | Novus (NB100-2284)) |
| Alexa Fluor 488 | IHC | Donkey anti rabbit IgG | 1:100 | Invitrogen (A21206) |
| Alexa Fluor 488 | IHC | Donkey anti goat IgG | 1:100 | Invitrogen (A11055) |
| Alexa Fluor 568 | IHC | Donkey anti mouse IgG | 1:100 | Invitrogen (A10037) |
| Alexa Fluor 568 | IHC | Donkey anti goat IgG | 1:100 | Invitrogen (AA21432) |
| Alexa Fluor 568 | IHC | Donkey anti rabbit IgG | 1:100 | Invitrogen (A10042) |

**Supplementary Table 1. Primary and secondary antibodies used for ICC and IHC.**
