# Supplementary material for: Cardiovascular ACE2 receptor expression in patients undergoing heart transplantation
Source: ESC Heart Fail. 2021 Aug 12;8(5):4119–29. doi: 10.1002/ehf2.13528 (PMC8497226; doi:10.1002/ehf2.13528)
Supplement: Supplementary file 3 — Table S2. Clinical variables significantly correlated with cardiac ACE2 expression or showing a non‐significant trend. Abbreviations: NT‐pro BNP, N‐terminal prohormone of brain natriuretic peptide; PCWP, pulmonary capillary wedge pressure; LVESD, left ventricular end‐systolic dimension; BSA, body surface area; CPEX, cardiopulmonary exercise testing; ECMO, extracorporeal membrane oxygenation; CABG, coronary artery bypass grafting; AF, atrial fibrillation. [file EHF2-8-4119-s001.docx]

|  | **Pearson** | ***P*-value** |
| --- | --- | --- |
| **Correlation with ACE2 in cardiomyocytes** |  |  |
| NT-pro BNP | 0.597 | 0.001 |
| PCWP | 0.371 | 0.043 |
| LVESD | 0.502 | 0.057 |
| Age | -0.341 | 0.042 |
| Weight | -0.447 | 0.007 |
| BSA | -0.532 | 0.005 |
| Diabetes Mellitus | -0.432 | 0.009 |
| **Correlation with ACE2 in vasculature** |  |  |
| CPEX | 0.562 | 0.005 |
| ECMO | 0.322 | 0.055 |
| Previous CABG | 0.310 | 0.066 |
| AF | -0.338 | 0.044 |
| BSA | -0.342 | 0.087 |

**Supplementary Table 2. Clinical variables significantly correlated with cardiac ACE2 expression or showing a non-significant trend.**

Abbreviations: NT-pro BNP, N-terminal prohormone of brain natriuretic peptide; PCWP, pulmonary capillary wedge pressure; LVESD, left ventricular end-systolic dimension; BSA, body surface area; CPEX, cardiopulmonary exercise testing; ECMO, extracorporeal membrane oxygenation; CABG, coronary artery bypass grafting; AF, atrial fibrillation.
